# Supplementary figures and images for: Tac1 Deficiency Reduces the Severity of Enteric Bacterial Infection
Source: bioRxiv. 2025 May 30:2025.05.27.656414. Preprint. [Version 1] doi: 10.1101/2025.05.27.656414 (PMC12154658; doi:10.1101/2025.05.27.656414)

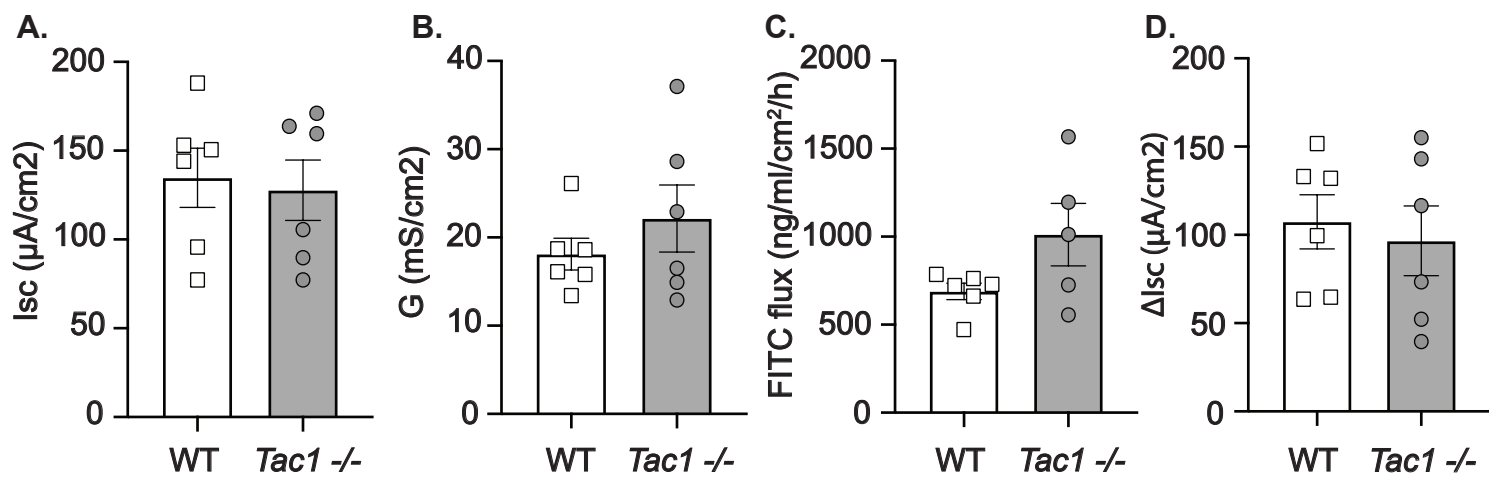

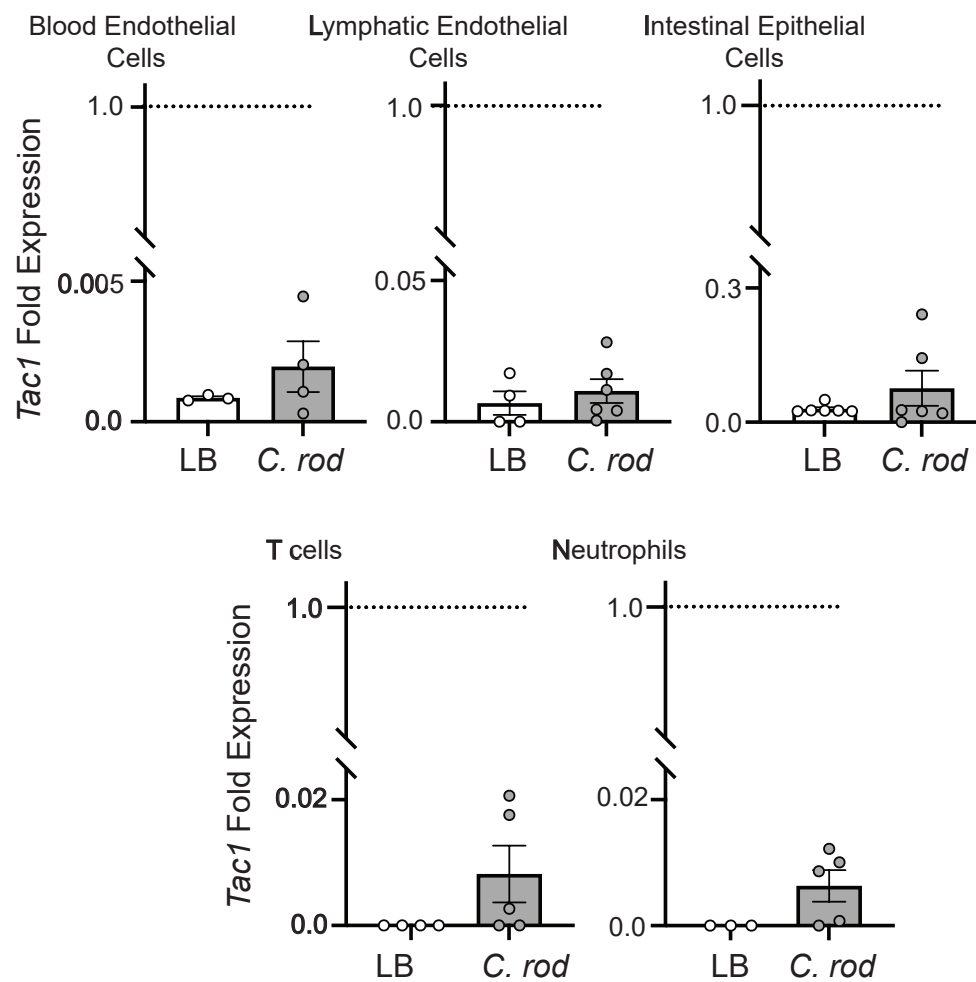

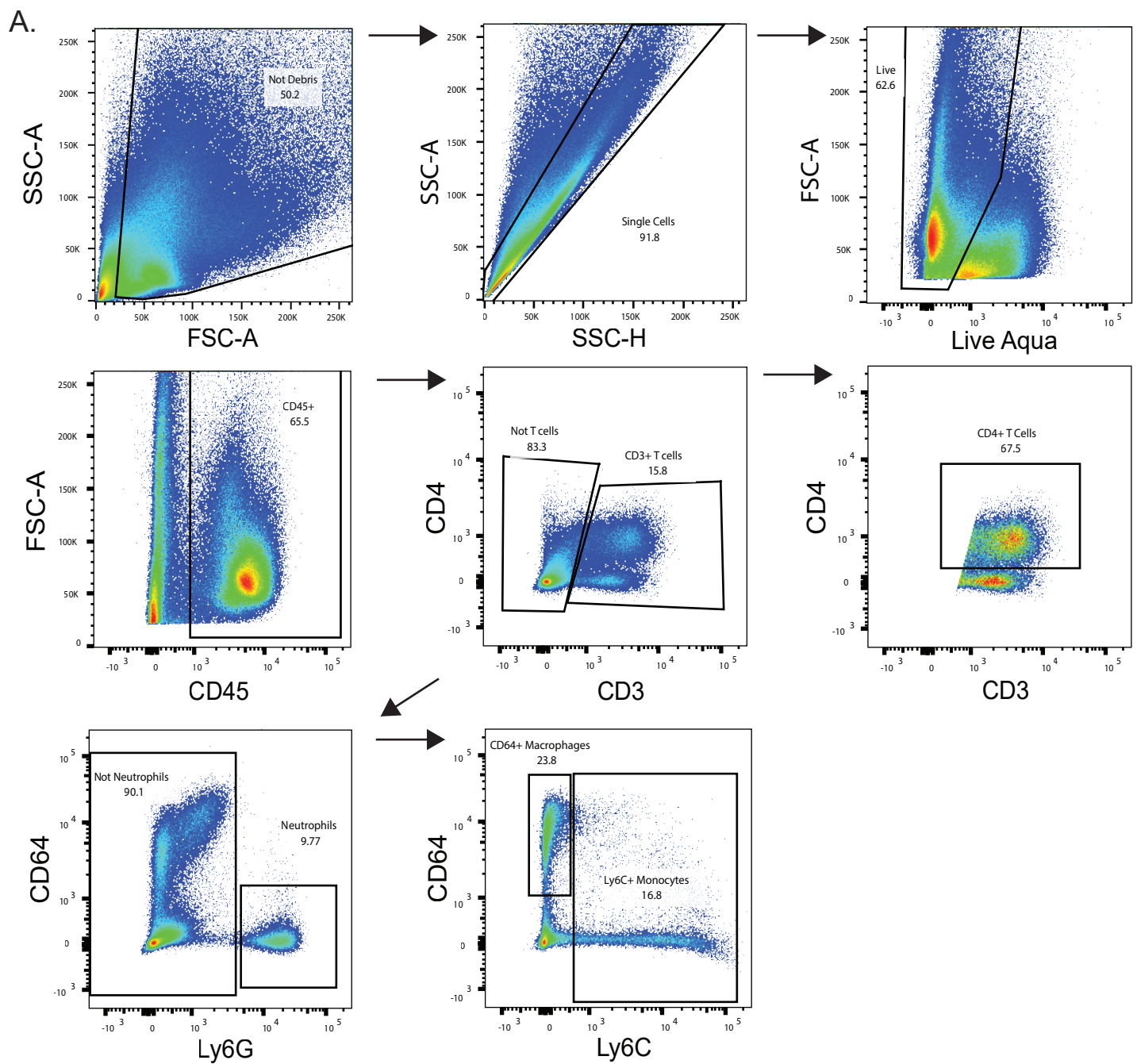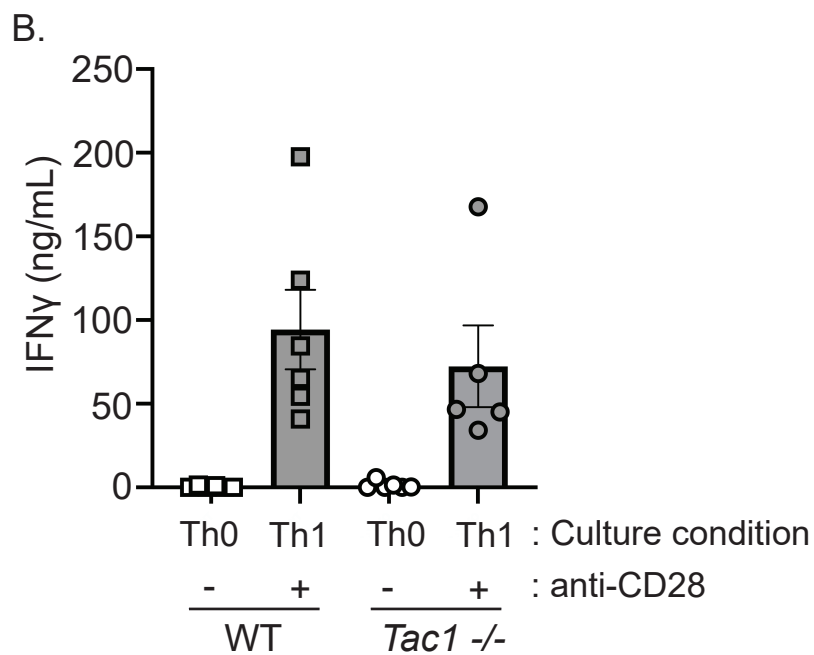

Supplement: Supplement 1 — Figure S1. Assessment of intestinal physiology in Tac1−/− mice. Colonic physiology including short circuit current “Isc” (A), conductance “G” (B), macromolecular permeability (C), and evoked Isc response to carbachol (D) in uninfected WT and Tac1−/− mice was assessed by Ussing chamber. Data are presented from individual mice with mean ± SD, Student’s two-tailed T-test. Figure S2. Tac1 expression in FACS-sorted cell populations. Single cell suspensions from the colon of uninfected and infected WT mice (10 days post-infection) were subjected to FACS followed by qPCR to detect Tac1 mRNA expression in blood endothelial cells, lymphatic endothelial cells, T-cells, neutrophils, and other immune cells (CD45+ CD3− Ly6G−). Expression normalized to Tac1 expression from WT dorsal root ganglia. Intestinal epithelial cells were obtained by agitation in EDTA-containing media. Data are presented from individual mice with mean ± SD, *P<0.05, ** P< 0.005, ***P<0.001, one-way ANOVA followed by post-hoc analysis with Tukey’s multiple comparison test. Figure S3. Flow cytometry gating analysis and in vitro T-cell differentiation assays. Gating strategy used to enumerate the indicated cell populations (A) from the colon of uninfected or 10 days post-C. rodentium infection in WT or Tac1−/− mice. The ability of mesenteric lymph node T-cells from Tac1−/− mice or WT to differentiate and produce IFNγ was assessed by ELISA following in vitro Th1 differentiation and stimulation (B). Data are presented from individual mice with mean ± SD, *P<0.05, ** P< 0.005, ***P<0.001, one-way ANOVA followed by post-hoc analysis with Tukey’s multiple comparison test. [file media-1.pdf]
